# Hyperthyroidism and clinical depression: a systematic review and meta-analysis

**DOI:** 10.1038/s41398-022-02121-7

**Published:** 2022-09-05

**Authors:** Henry Bode, Beatrice Ivens, Tom Bschor, Guido Schwarzer, Jonathan Henssler, Christopher Baethge

**Affiliations:** 1 Department of Psychiatry and Psychotherapy, Faculty of Medicine, University of Cologne, Cologne, Germany; 2 Department of Psychiatry and Psychotherapy, Faculty of Medicine, Technical University of Dresden, Dresden, Germany; 3 Institute of Medical Biometry and Statistics, Faculty of Medicine and Medical Center, University of Freiburg, Freiburg, Germany; 4 Department of Psychiatry and Psychotherapy, Charité Universitätsmedizin Berlin, Berlin, Germany

**Keywords:** Depression, Pathogenesis

## Abstract

Hyperthyroidism and clinical depression are common, and there is preliminary evidence of substantial comorbidity. The extent of the association in the general population, however, has not yet been estimated meta-analytically. Therefore we conducted this systematic review and meta-analysis (registered in PROSPERO: CRD42020164791). Until May 2020, Medline (via PubMed), PsycINFO, and Embase databases were systematically searched for studies on the association of hyperthyroidism and clinical depression, without language or date restrictions. Two reviewers independently selected epidemiological studies providing laboratory or ICD-based diagnoses of hyperthyroidism and diagnoses of depression according to operationalized criteria (e.g. DSM) or to cut-offs in established rating scales. All data, including study quality based on the Newcastle-Ottawa Scale, were independently extracted by two authors. Odds ratios for the association of clinical depression and hyperthyroidism were calculated in a DerSimonian-Laird random-effects meta-analysis. Out of 3372 papers screened we selected 15 studies on 239 608 subjects, with 61% women and a mean age of 50. Relative to euthyroid individuals, patients with hyperthyroidism had a higher chance of being diagnosed with clinical depression: OR 1.67 ([95% CI: 1.49; 1.87], *I*^2^: 6%; prediction interval: 1.40 to 1.99), a result supported in a number of sensitivity and subgroup analyses. The OR was slightly less pronounced for subclinical as opposed to overt hyperthyroidism (1.36 [1.06; 1.74] vs. 1.70 [1.49; 1.93]). This comorbidity calls for clinical awareness and its reasons need investigation and may include neurobiological mechanisms, common genetic vulnerability and a generally heightened risk for clinical depression in patients with chronic somatic disorders.

## Introduction

A link between thyroid disorders and depression has been investigated for decades [1]. Researchers uncovered several possible interactions between thyroid metabolism, the HPT-Axis and mood regulation [2–6], and the association of hypothyroidism with depression has been the focus of various meta-analyses [7–9]. They yielded positive results, but the extent of the comorbidity may have been overestimated [10]: In the most recent meta-analysis, we estimated an odds ratio for hypothyroidism and clinical depression of 1.30 [1.08–1.57] in population-based studies [11].

The association of depression with the other end of the thyroid disorder spectrum, hyperthyroidism, has been much less investigated, although it consists of a common group of conditions [12]. For example, NHANES III found general population prevalences of 0.7% and 0.5% for subclinical and overt hyperthyroidism, respectively [13]. A Danish register-based study [14] found that subjects with hyperthyroidism had a higher likelihood of developing depression than euthyroid controls (Hazard Ratio (HR): 1.54 [1.36–1.74]). Similarly, Williams et al. [15] found serum T4 to be positively associated with depression. In contrast, an individual patient data meta-analysis of six studies by Wildisen et al. [16] yielded no relevant association of subclinical hyperthyroid states and depression.

So far, no clear picture of a link between hyperthyroidism and depression emerged. Therefore, we conducted a systematic review and meta-analysis of studies presenting data on established hyperthyroidism—either subclinical or overt—and clinically relevant depression. To reduce bias, we restricted this meta-analysis to epidemiological and population-based studies and did not include studies based on samples from outpatient departments for thyroid or mood disorders.

## Methods

This is a systematic review and meta-analysis registered in PROSPERO (CRD42020164791). Its reporting is based on the PRISMA 2020 and MOOSE guidelines [17, 18].

### Data sources and searches

We conducted a systematic search in MEDLINE and PubMed Central via PubMed, in PsycINFO via EBSCOhost, and in Embase to identify epidemiological studies on the association of hyperthyroidism and depression (last update on May 4, 2020). We combined generic terms for depression, hyperthyroidism, and population-based study designs (Supplementary Methods).

### Study selection

***Inclusion criteria***

1. Study design: Cohort and cross-sectional studies.
2. Study population: Studies needed to be representative of the general population. If study groups were not randomly sampled from the general population, studies were only eligible if they (1) drew on very broad and diverse populations, such as civil servants or the totality of hospitalized patients in one country, and (2) study reports were not suggestive of biases, i.e. included a complete report of the recruitment and selection process.
3. Exposure: Hyperthyroid thyroid disorders, either subclinical, overt or, if nothing else was stated, of autoimmune origin (i.e. Graves’ Disease, but not Hashimoto thyroiditis). Disorders needed to be diagnosed by established laboratory methods or had to be drawn from registers employing data with documented reliability.
4. Outcome: Clinically significant depression, either defined as major depressive disorder (MDD) diagnosis according to established diagnostic systems, e.g. DSM or ICD, or an above-threshold score in established psychopathology rating scales for depression [19], as specified by study authors. Diagnoses could originate with assessment rating scales, standardized interviews (e.g. WHO-CIDI) or from registers including hospital data with documented reliability. To err on the conservative side we did not associate hyperthyroidism with any change in depression scores, because, in the general population, variations in depression scale scores *below* a predefined cut-off point for caseness are not indicative of clinical depression and may create pseudo-effects.

***Exclusion criteria***

1. Study design: Case-control studies

### Data extraction and quality assessment

Titles and abstracts retrieved in the literature search were independently screened by two authors (HB, BI). “Grey” literature was included and no language or date restrictions applied. We searched bibliographies of every article eventually included. All articles potentially eligible were read independently by two authors (HB, BI), and data of included studies were extracted independently by two authors (HB, BI) using an Excel-based standardized data extraction form in accordance with the Cochrane Collaboration Handbook [20]. Disagreements were solved by discussion with the senior author (CB). If no effect sizes or sufficient data for calculation were reported, we contacted authors by e-mail.

All studies included were rated independently by two authors (HB, BI) for their risk of bias, using the Newcastle-Ottawa Scale (NOS) adaptation for cohort [21] and cross-sectional studies [22]. To be rated as “low risk of bias”, studies needed to be categorized in the highest NOS-category, i.e. they needed to receive all or all but one star in the rating system.

### Data synthesis and analysis

To account for differences in study settings and methodology we used random effects analyses (DerSimonian & Laird) [23]. Statistical heterogeneity is reported as *I*^2^ statistic and Tau (τ). We assessed publication bias in funnel plots and Egger’s test [24] and estimated the role of missing studies in trim-and-fill-analyses [25]. Prediction intervals were calculated to account for the heterogeneity between studies [26]. We conducted leave-one-out analyses if forest plots indicated a disproportionate effect of single studies. All calculations have been carried out in Comprehensive Meta-Analysis (CMA) Version 3 [27] and R [28], using the packages meta [29] and metafor [30].

#### Primary outcome analysis

The primary outcome is the association of hyperthyroidism and clinical depression. We compared depression prevalence of patients with versus without hyperthyroidism and expressed the results as odds ratio (OR) ± 95% confidence interval (CI). If studies reported effects as risk ratio (RR) or hazard ratio (HR), we transformed these effects into ORs (Supplementary Methods). If studies reported multiple differently adjusted effect sizes, we included those with minimum adjustment to be as coherent as possible with unadjusted or self-calculated effect sizes. The analysis includes all studies reporting results for overt and/or subclinical hyperthyroidism. If a study reported effects for both, only results for overt hyperthyroidism were included to maintain independence. Calculations and formulae are listed in the supplementary information (Supplementary Methods).

#### Subgroup analyses

We subdivided hyperthyroidism into both its overt and subclinical form, as defined in the studies included, and stratified our primary analyses by gender, risk of bias, intake of thyroid medication, a core group of strictly population-based studies, and assessment of depression. To minimize bias, the subgroup analysis investigating gender-specific effects was conducted only on studies that reported effects for both genders. All of the above stratifications were also repeated in the subgroup analyses of overt and subclinical hyperthyroidism.

#### Post hoc analyses

We compared studies on older populations (age ≥ 60 years) with studies on subjects of all ages to detect age-specific trends. To compare the effects of hyperthyroidism with those of hypothyroidism, we selected studies reporting an effect for both thyroid disorders and analysed them separately.

## Results

**Fig. 1:**
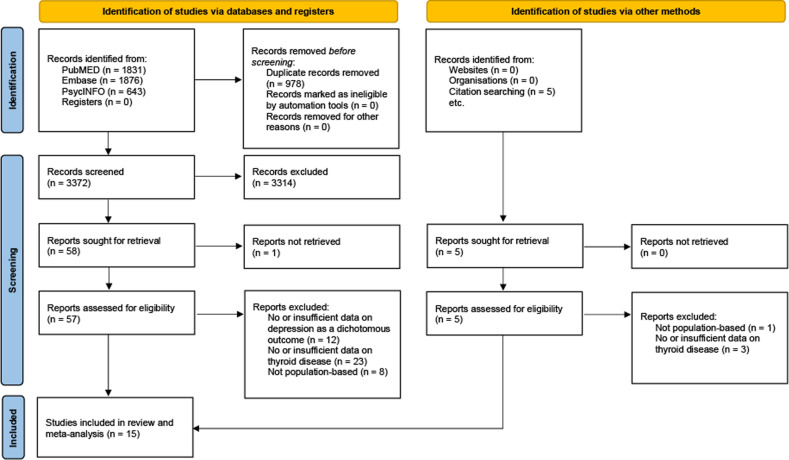
PRISMA flow diagram showing the study selection process.

Our search yielded 4350 articles. After exclusion of duplicates, 3372 were screened and out of those, 62 were assessed for eligibility in full text. Fifteen studies [31–45] are included in this meta-analysis (PRISMA flowchart, Fig. 1). Three studies reported effects for overt, 7 for subclinical and 4 for both types of hyperthyroidism. The study of Chen et al. [33] reported effects for Grave’s disease, which we considered a plausible proxy of overt hyperthyroidism.

Table 1 displays characteristics of all studies: Three cohort (72.5% of participants) and 12 cross-sectional studies (27.5%) accounted for a total of 239,608 participants, ranging from 60 [41] in the smallest to 150,960 [44] in the largest study. Study size weighted mean age of participants was 50 years. The overall proportion of women was 60.8%.

**Table 1:** Characteristics of studies included in the present meta-analysis.

Study	Study type	*N*	NOS	Sample	Gender	Age range	Assessment of thyroid disorder	Assessment of depression
Almeida et al. [31]	Cross-Sectional	3504	10+	Subclinical	Male	69–87	fT4, TSH	Diagnosis
Bensenor et al. [32]	Cross-Sectional	12630	7	Subclinical	Mixed	35–74	fT4, TSH	Diagnosis
Chen et al. [33]	Cohort	20975	9+	Overt	Mixed	≥ 20	Register	Register
					Female			
					Male			
De Jongh et al. [34]	Cross-Sectional	1120	7	Subclinical	Mixed	≥ 65	fT4, TSH	CES-D
Engum et al. [35]	Cross-Sectional	28830	9+	Overt	Mixed	40–89	fT4, TSH	HADS-D
				Subclinical	Mixed			
Hong et al. [36]	Cross-Sectional	1704	8	Subclinical	Mixed	19–76	fT4, TSH	PHQ-9
Ittermann et al. [37]	Cohort	1718	8+	Overt	Mixed	20–79	TSH	Diagnosis
Kim et al. [38]	Cross-Sectional	458	8	Subclinical	Mixed	≥ 65	TSH	GMS-B3
Kvetny et al. [39]	Cross-Sectional	13521	8	Subclinical	Mixed	≥ 20	TSH	Diagnosis
					Female			
					Male			
Manciet et al. [40]	Cross-Sectional	399	7	Overt	Mixed	≥ 65	fT4, TSH	CES-D
				Subclinical	Mixed			
Maugeri et al. [41]	Cross-Sectional	60	6	Overt	Mixed	≥ 70	T3, T4, TSH	GDS-30
Pop et al. [42]	Cross-Sectional	558	8	Overt	Female	47–54	fT4, TSH	EDS
				Subclinical	Female			
Shinkov et al. [43]	Cross-Sectional	2287	7	Subclinical	Mixed	20–84	TSH	Zung SDS

+ = included in the RoB-analysis.

*CES-D* Center for Epidemiologic Studies Depression Scale, *HADS-D* Hospital Anxiety and Depression Scale, *PHQ-9* Patient Health Questionnaire 9, *GMS-B3* Geriatric Mental State Diagnostic Schedule, *GDS-30* Geriatric Depression Scale 30, *EDS* Edinburgh Depression Scale, *Zung SDS* Zung Self-Rating Depression Scale, *BDI-Ia* Beck Depression Inventory Ia, *Diagnosis* DSM- or ICD-conforming diagnosis of depression; *NOS* Newcastle-Ottawa Scale.

Six studies reported depression in DSM- or ICD-conforming diagnoses of major depressive disorder. Cut-off points in depression scores were employed in 9 studies.

In 13 studies, authors provided diagnoses of thyroid disorders based upon established laboratory methods, 2 used register data on ICD-based diagnoses. Intake of thyroid or anti-thyroid medication was allowed in 10 studies.

Applying the Newcastle-Ottawa Scale, 3 cohort and 2 cross-sectional studies were rated as carrying a “low risk of bias”.

Full data describing all included studies are presented in the supplementary information (Supplementary Table 1).

**Fig. 2:**
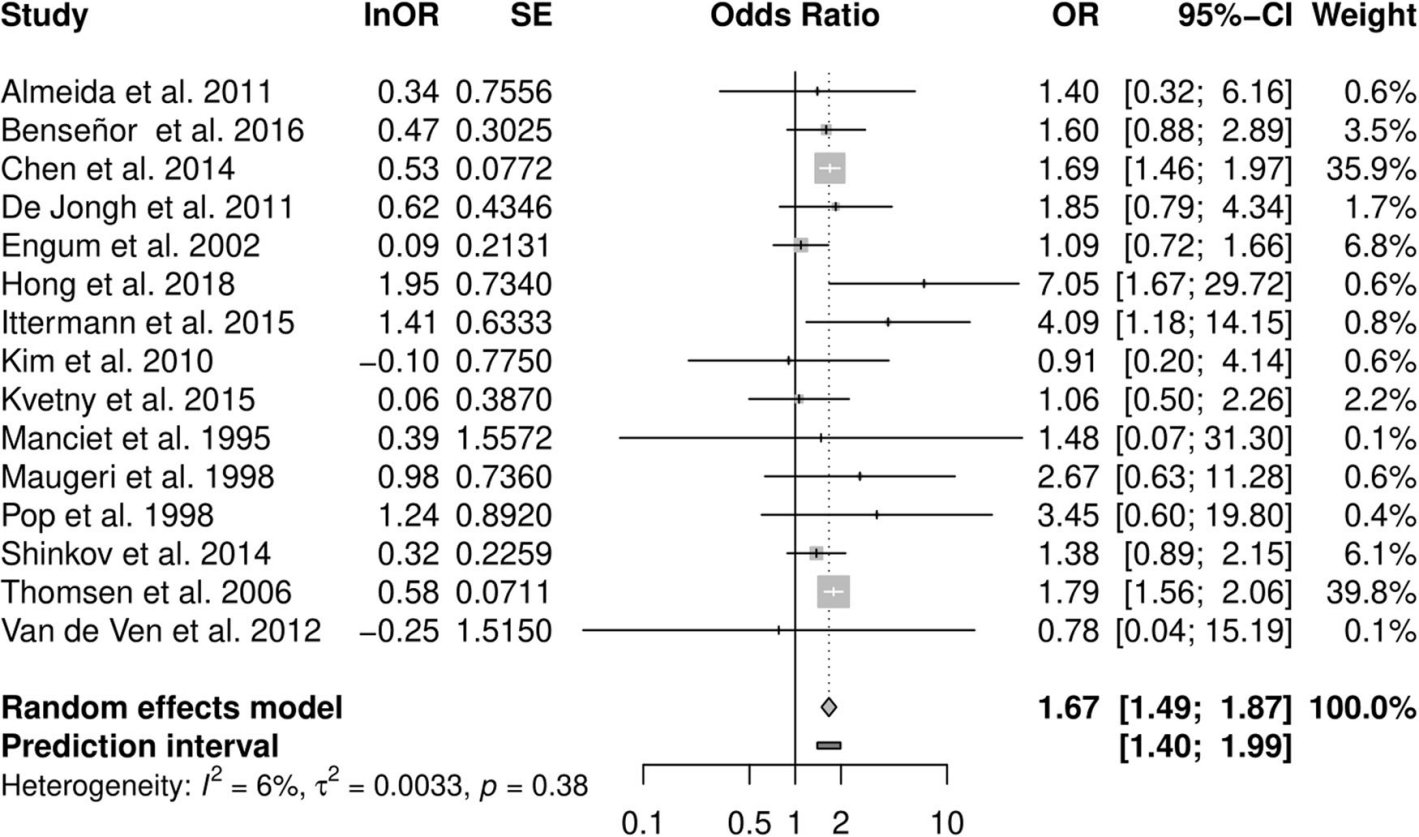
Association of hyperthyroidism and depression. Forest plot of the primary analysis on the association of hyperthyroidism and depression. Odds ratios greater than 1 indicate a stronger association of depression with hyperthyroidism than with euthyroidism, odds ratios smaller than 1 indicate a weaker association.

Pooled analysis of all studies resulted in an OR estimate of 1.67 [1.49–1.87] of clinical depression in all types of hyperthyroidism relative to euthyroidism (Fig. 2 and Table 2), no relevant heterogeneity was observed (*I*² = 6.4%, τ = 0.058; prediction interval: 1.40 to 1.99).

**Table 2:** Main results.

Analysis	Odds ratio	Heterogeneity	Prediction Interval	Egger’s Test
Primary Outcome	1.67 [1.49–1.87], *p* < 0.001, *N* = 15	*I*² = 6.4%, τ = 0.058	1.40–1.99	*p* = 0.964
Low RoB	1.66 [1.40–1.97], *p* < 0.001, *N* = 5	I² = 41.6%, τ = 0.114	1.05–2.62	
Overt	1.70 [1.49–1.93], *p* < 0.001, *N* = 8	*I*² = 12.9%, τ = 0.069	1.34–2.14	
Subclinical	1.36 [1.06–1.74], *p* = 0.015, *N* = 11	*I*² = 13.4%, τ = 0.151	0.87–2.12	*p* = 0.262
Female	1.37 [0.91–2.05], *p* = 0.130, *N* = 3	*I*² = 46.7%, τ = 0.254	0.02–87.42	
Male	1.84 [1.34–2.54], *p* < 0.001, *N* = 3	*I*² = 0%, τ = 0.000	0.23–14.81	
	*Post-hoc*			
Hypothyroidism	1.36 [1.02–1.82], *p* = 0.037, *N* = 14	*I*² = 63.8%, τ = 0.373	0.57–3.27	*p* = 0.711
Hyperthyroidism	1.61 [1.34–1.93], *p* < 0.001, *N* = 14	*I*² = 13.0%, τ = 0.124	1.14–2.25	*p* = 0.973

Effects are reported as OR and 95% confidence interval. N describes the number of studies included in the analysis. Egger's *p*-value is reported as two-sided, values <0.1 indicate potential publication bias. For reasons of power, Egger’s test was only carried out if at least 10 studies were included in the analysis.

Additional results can be obtained from the supplementary information.

Subgroup analysis of studies restricted to overt hyperthyroidism resulted in a similar OR of 1.70 [1.49–1.93], and subclinical hyperthyroidism was also associated with depression (1.36 [1.06–1.74], Table 2). Stratification by gender, based on results from three studies, revealed an OR of 1.37 [0.91–2.05] among women and 1.84 [1.34–2.54] in men (p-value between effects: 0.257). Strictly population-based studies as well as studies that allowed for thyroid or anti-thyroid medication reported slightly weaker summary associations with depression. A cohort study design and a DSM- or ICD-conforming diagnosis of depression resulted in moderately stronger effects (Supplementary Table 2.1). Stratifying subgroup analyses in overt and subclinical hyperthyroidism yielded similar effects (Supplementary Table 2.2).

The funnel plot of the primary analysis indicated an asymmetric reporting of effects, with studies missing in the lower right quadrant, indicating the possibility of biased reporting in favour of *weaker* effects. However, Egger’s test was not positive.

Analysing studies with low risk of bias confirmed the results of the primary subgroup analyses on overt hyperthyroidism, while the association of subclinical hyperthyroidism with depression became negligible (1.08 [0.8–1.46]).

In a leave-one-out analysis, study weight was distributed unevenly with two studies carrying almost 80% of the weight. Exclusion of these large register studies by Chen et al. [33] and Thomsen et al. [44] slightly lowered the association (1.61 [1.34–1.93] and 1.58 [1.35–1.85] respectively). In subclinical hyperthyroidism, removal of the study by Engum et al. [35] increased ORs to 1.52 [1.16–1.99].

Similar to the primary analysis, heterogeneity was low or moderate in subgroup and sensitivity analyses, with *I*² generally not exceeding 50% (Supplementary Table 2).

Post-hoc analyses on studies on older populations reported effects similar to those of studies on all ages (Supplementary Table 2).

In fourteen studies effects for both, hyper- and hypothyroidism were reported [31–45]. Thomsen et al. published corresponding data on hypothyroidism in a separate study [46]. Combining studies on overt and subclinical disease resulted in a nominally weaker association of depression with hypothyroidism (1.36 [1.02–1.82]]) than with hyperthyroidism (1.61 [1.34–1.93]). In overt hyperthyroidism (1.66 [1.22–2.26]) associations were similar to those in overt hypothyroidism (1.69 [0.83–3.45]). Subclinical disease showed equal ORs in hyper- (1.36 [1.06–1.74]) and in hypothyroidism (1.35 [1.05–1.73]). After correction for potentially missing studies (Egger’s test *p*-value = 0.003) however, the association of hypothyroidism with depression decreased to an OR of 1.14 [0.88–1.47].

## Discussion

To our knowledge, this is the first meta-analysis investigating both subclinical and overt hyperthyroidism and its association with clinical depression in the general population. We found a statistically significant association of hyperthyroidism and depression, with small confidence as well as prediction intervals indicating a robust effect. The results are supported by a variety of subgroup as well as sensitivity analyses and by low heterogeneity. Therefore, even though fewer studies have been carried out on hyper- than on hypothyroidism (OR: 1.30 [1.08–1.57]) [11], we consider the evidence of an association with clinical depression and the effect itself marginally stronger in hyperthyroidism (OR 1.67 [1.49–1.87]).

These findings challenge earlier research on hyperthyroidism and depression: Wildisen et al. [16], for example, reported no relevant effect of subclinical hyperthyroidism on BDI-scores. However, they did not analyse patients with overt hyperthyroidism. Of note, more in line with Wildisen and co-authors’ study, our results point to a weaker, if any, association of subclinical hyperthyroidism with clinical depression than in overt hyperthyroidism.

This gradient in effect size may support the notion that it is not primarily autoimmunity that drives the association but possibly the increase in thyroid hormones. Further, in an earlier meta-analysis we could not support an association of TPO-antibody positivity with clinical depression [11]. At the pathophysiological level, the findings are consistent with several hypotheses of neuroendocrine causes of depression: Dysbalanced, thyroid hormones as key regulators of metabolism can contribute to typical symptoms of depression such as sleep disturbance, weight change, fatigue or psychomotor agitation [47]. They also stimulate cortical 5-HT secretion [48] and might act as co-transmitters in the noradrenergic system [49], influencing monoaminergic transmission in the brain. Several animal models showed that induction of hypo- and hyperthyroid states in rats significantly altered cortical monoamine levels: While in hypothyroidism 5-HT levels decreased in multiple brain regions [50–52], in hyperthyroidism, norepinephrine concentration as well as the number of 5-HT2-receptors were downregulated simultaneously with an increase in 5-HT levels [50, 52–54]. One study reported depression-like behaviour in rats with induced states of both hypo- and hyperthyroidism [55]. Interestingly, hyperthyroid rats also showed anxiety-like behaviour. Further, both hypo- as well as hyperthyroid disturbances of the HPT-axis can lead to hypercortisolism [56–60], which is often found in patients suffering from depression [6, 61–63]. Possibly, inflammation provides a link between thyroid disorders and depression, because with Hashimoto and Graves‘ disease, two of the leading causes of thyroid dysfunction result from autoantibodies against thyroid tissue, are characterized by lymphocytic infiltrates and increased levels of proinflammatory cytokines (such as IL-6 and TNF-alpha), and may have their origins in viral infections [64, 65]. Recently, similar factors have been discussed in the etiology of depression [66], and common pathways may explain the comorbidity described in primary studies and in this meta-analysis.

In principle, the association between thyroid disorders and depression may also be based on a common genetic vulnerability. Some structures involved in brain thyroid hormone metabolism, such as the deiodinase enzymes (DIO) type 1,2 and 3 or the thyroid hormone transporter OATP1C1, may lead to a local state of hormone deficiency when functionally impaired or overactive, facilitating the development of depression potentially regardless of serum thyroid hormone levels [4, 5, 67, 68]. Studies showed that some DIO2 polymorphisms were indeed associated with worse psychological well-being [69] and DIO2 expression was reduced in patients suffering from recurrent depressive disorders (rDD) [70]. DIO1 variants were associated with lifetime MDD in Caucasian female individuals [71] and DIO1 expression was also found to be decreased in subjects with rDD [72]. However, other studies could not link DIO expression or certain polymorphisms to depression or impaired well-being [73, 74]. Variation of the OATP1C1 gene was connected to fatigue and depression in hypothyroid individuals [75] and to depression in subjects who suffered from ischemic stroke [76]. We are not aware, however, of studies investigating the association of depression with genetic variations leading to a brain-specific local state of hyperthyroidism.

In an entirely different approach, the association of hyperthyroidism and clinical depression may be explained by the observation that chronic conditions as such are often related to a greater risk of being depressed [77–80]. In this framework, a chronic condition, for example, hyperthyroidism, acts as a stressor and may, particularly in vulnerable people, contribute to the development of clinical depression.

Reverse causation also needs consideration: Subsequent to the elevation of cortisol caused by depression, TRH production might be stimulated and lead to an overproduction of T4 [2, 5]. Normalization or, rather, a decrease of T4 levels after successful treatment of depressive disorder has also been observed [81, 82]. In general, however, the common paradigm in depression research is one of interdependence, not of one factor causing the other [83, 84], for example, with regard to the heightened cardiovascular risk of patients with depression.

A hint towards the predominant direction of the effect responsible for the observed association might be found in our subgroup analyses. Here, studies were stratified by study design, with cohort studies that assured absence of depression at baseline showing stronger effects than cross-sectional studies. While this can be understood as a sign for a stronger effect of hyperthyroidism on depression than vice versa, the analysis was only based on three cohort studies and therefore has to be regarded as preliminary.

A key feature of the present study is its focus on clinical depression. As a result, we may have missed subtle changes in psychopathology. However, not only was there no indication of such an effect in Wildisen and co-authors’ study [16], but in searching for differences in low and subclinical score ranges lies the risk of inflating small findings of doubtful clinical relevance. The problem of employing subclinical phenomena works also in thyroid parameters: Williams et al. [15], in their early meta-analysis, took into account the full range of thyroid hormones, physiological and pathological alike. Interestingly, however, even with their very broad approach they arrived at no stronger association than the one in the present study.

At the same time, relying on clinical data derived only from routine examinations in outpatient clinics for mood or metabolism disorders introduces selection bias [10], hence our restriction to epidemiological studies. In our view, therefore, the OR presented in this study represents a conservative estimate of the association. Since the effect we estimated is moderate it is worth noting that relatively small effect sizes are common in medicine, even in established biomarkers [85].

With regard to clinical practice, our results suggest that heightened awareness of depression is justified in patients with hyperthyroidism, as is TSH screening among patients with depression. In distinction to our analysis of hypothyroidism and depression—where the observed ORs were 0.71 [0.40–1.25] and 1.48 [1.18–1.85] for men and women respectively [11]—we did not find a clear-cut gender differential, although men with hyperthyroidism were slightly more affected by clinical depression than women. In fact, for women, our results are inconclusive, as the confidence interval includes a null effect. This applies all the more to the findings regarding women with subclinical hyperthyroidism where no association is apparent. Of note, the data were not sufficient for a subgroup analysis on ethnic differences indicating a need for future research.

In regard to therapy, both conditions, hyperthyroidism and depression, demand guideline-oriented treatment. We are not aware of an established treatment that would target the two diseases in one approach.

Hyperthyroidism is not as prevalent as hypothyroidism in the general population. Assuming a population of 332.5 million people in the US [86], a hyperthyroidism prevalence of 1.3% [13], and a 12-months depression prevalence of 6.7% [87], an OR of 1.67 translates into about 484 thousand people with the comorbidity, 194 thousand of those presumably due to hyperthyroidism. Provided that there are 22.3 million people with depression each year, in a model assuming that hyperthyroidism causes depression, hyperthyroidism contributes about 0.9% to the pandemic of depression.

We focussed on population-based studies, but the term leaves some room for debate: For example, a sample of civil servants, such as the one investigated by Benseñor et al. [32], is not population-based in the strict sense. However, while it is likely that prevalence differs contingent on the sample, it seems implausible that the association between hyperthyroidism and depression differs meaningfully in such a large and diverse group of people. Reassuringly, sensitivity analysis restricted to strictly population-based studies yielded no substantially different results (Supplementary Table 2). In the same vein, exclusion of register studies did not substantially change the summary estimate.

In conclusion, there is an association of hyperthyroidism with clinical depression (1.67 [95% CI: 1.49–1.87]), that is stronger in overt than in subclinical hyperthyroidism, pointing to a possibly biological association of both conditions. This should raise awareness in clinicians and researchers alike: Not only hypothyroid but also, and especially, hyperthyroid patients are at higher risk for depressive disorders and should be monitored for signs of clinical depression. How a hyperthyroid metabolism influences mood is not yet explained and, particularly regarding sex, deserves greater attention in the future research of thyroid–brain interactions.

## Supplementary information

Supplemental Material
