# Supplementary material for: Hyperthyroidism and clinical depression: a systematic review and meta-analysis
Source: Transl Psychiatry. 2022 Sep 5;12:362. doi: 10.1038/s41398-022-02121-7 (PMC9445086; doi:10.1038/s41398-022-02121-7)
Supplement: Supplementary file 1 — Supplemental Material [file 41398_2022_2121_MOESM1_ESM.docx]

**Supplementary Information**

**Table of Contents:**

- **Supplementary Methods:** Search Criteria; Calculations
- **Supplementary Table 1:** Included Studies
- **Supplementary Table 2.1:** Additional Results Primary Analysis
- **Supplementary Table 2.2:** Additional Results Overt and Subclinical Hyperthyroidism
- **References**

**Search Criteria:** Structured search queries used in the literature search. Search queries for EBSCOhost and Embase were identical.

**Calculations:** Calculations used in the statistical analysis, mainly effect size transformation. For the complete statistical procedure please see the corresponding section of the main article.

**Supplementary Table 1:** Additional information on the included studies, including the respective risk of bias assessment and effect size. Given information was used in data analysis (e.g. for stratification) and can be used to reproduce all results reported in the meta-analysis.

**Supplementary Table 2.1 and 2.2:** Additional results obtained from the meta-analysis, collectively referred to as “Supplementary Table 2” in the main publication.

**Supplementary Methods:**

**Search Criteria**

The database search was performed in three separate databases: Medline (via PubMed), PsycINFO (via EBSCO host) and Embase. Since all databases require a different syntax, different search queries were created.

*PubMed:*

(Hashimoto*[Text Word] OR "Grave* Disease"[Text Word] OR AIT[Text Word] OR Basedow[Text Word] OR "Ord* Disease"[Text Word] OR thyroid*[Text Word] OR hypothyroid*[Text Word] OR hyperthyroid*[Text Word] OR Hypothyroidism[MeSH Terms] OR Hyperthyroidism[MeSH Terms] OR "Thyroiditis, Autoimmune"[MeSH Terms] OR "Hashimoto Disease"[MeSH Terms] OR "Graves Disease"[MeSH Terms] OR Thyroid diseases[MeSH Terms] OR Thyroiditis[MeSH Terms] OR "Postpartum Thyroiditis"[MeSH Terms] OR "Thyroiditis, subacute"[MeSH Terms] OR "Antibodies, Thyroid-Stimulating"[MeSH Terms])

AND

(Depress*[Text Word] OR affective[Text Word] OR "Mood Disorder*"[Text Word] OR dysthym*[Text Word] OR cyclothym*[Text Word] OR mania[Text Word] OR manic[Text Word] OR hypomani*[Text Word] OR "unipolar disorder"[Text Word] OR bipolar[Text Word] OR "abnormal mood"[Text Word] OR melancholia[Text Word] OR mood[Text Word] OR "MADRS"[Text Word] OR "HAM-D"[Text Word] OR "Depressive disorder"[MeSH Terms] OR "cyclothymic disorder"[MeSH Terms] OR "Bipolar Disorder"[MeSH Terms] OR "Mood disorder"[MeSH Terms] OR "Quality of life"[MeSH Terms] OR "Affective symptoms"[MeSH Terms] OR "Bipolar and Related Disorders"[MeSH Terms])

AND

(epidemiolog*[Text Word] OR cohort[Text Word] OR random*[Text Word] OR cross-sectional[Text Word] OR population[Text Word] OR prospective[Text Word] OR "Epidemiologic Studies"[MeSH Terms] OR "Cohort Studies"[MeSH Terms] OR "Cross-Sectional Studies"[MeSH Terms] OR epidemiology[MeSH Terms] OR "Depressive disorder/epidemiology"[MeSH Terms] OR "Hypothyroidism/epidemiology"[MeSH Terms] OR "Hyperthyroidism/epidemiology"[MeSH Terms] OR "Mental disorders/epidemiology"[MeSH Terms] OR "Metabolic diseases/epidemiology"[MeSH Terms] OR "Depression/epidemiology"[MeSH Terms])

FILTER: SPECIES: Human

*EMBASE / EBSCOhost:*

(Hashimoto* OR "Graves Thyroiditis" OR "Graves Disease" OR AIT OR Basedow OR "Ord* Thyroiditis" OR "Ord* Disease" OR thyroid* OR hypothyroid* OR hyperthyroid*)

AND

(Depress* OR affective OR "Mood Disorder*" OR dysthym* OR cyclothym* OR mania OR manic OR hypomani* OR "unipolar disorder" OR bipolar OR "abnormal mood" OR melancholia OR mood OR "MADRS" OR "HAM-D")

AND

(epidemiolog* OR cohort OR communit* OR random* OR cross-sectional OR population OR prospective)

FILTER: SPECIES: Human

**Calculations**

**Transformation of Effect Sizes:** Studies included in this meta-analysis reported different types of effect sizes, including Odds Ratios (OR), Hazard Ratios (HR) and Risk Ratios (RR). To use these effect sizes in an analysis resulting in a pooled Odds Ratio (OR), we applied different approaches of transformation.

Transformation from RRs to ORs were based on this formula proposed by Zhang et al. 1998^1^,

$$OR=RR\times(\left( 1-r \right)+\left( r\times OR \right))$$

where „r“ is the incidence of the observed outcome in the non-exposed population.
We assumed that for depression, for which the incidence is reported to be 6.7% in the general US population^2^, the reported RRs would be almost equal to the respective ORs in the population. Thus, chances of introducing inaccuracy would be small.
However, to conduct an accurate transformation, we searched the respective studies and published literature for an incidence in the reference population and applied the formula accordingly.

Transformation of RRs to HRs were conducted using this formula proposed by Shor et al. 2017^3^,

$$RR=\frac{\left( 1-e^{HR\times\ln\left( 1-r \right)} \right)}{r}$$

which allows for a similar procedure. Again, studies and published literature were searched to find the incidence of depression in the reference population.

To validate the above made calculations, we used another approach presented by Altman and Andersen (1999)^4^. Based on a HR and the probability of survival “Surv.c”, Altman and Andersen proposed a way of calculating a number needed to treat (NNT). This approach can be adapted for ORs, resulting in the following formulae:

$$Surv.e={Surv.c}^{HR} ; RR=\frac{Surv.e}{Surv.c} ; OR=\frac{\frac{Surv.c}{\left( 1-Surv.c \right)}}{\frac{Surv.e}{(1-Surv.e)}}$$

Using probabilities of survival reported in the affected studies, we transformed HRs and RRs to ORs. This approach yielded the same results as the formulae above. We therefore consider the obtained ORs robust.

Manual calculation of ORs from raw study data was performed using standard formulae^5^.

Few studies reported asymmetric confidence intervals which were not processable by our software “Comprehensive Meta-Analysis V3” (CMA)^6^. To obtain a usable confidence interval, standard errors were calculated based on the reported OR and p-values or z-statistics. Confidence Intervals were then normalized around the reported OR.

**Supplementary Table 1:** Included Studies

| Study | Location | Name | Population | Design | N | NOS | Sample | Gender | Euthyroid [n] | Thyroid Disorder [n] | Female [%] | Age mean [y] | Age SD [y] | Age range [y] | Assessment Thyroid Disorder | Assessment Depression | Thyroid Medication Intake | OR | 95% CI |  |
| --- | --- | --- | --- | --- | --- | --- | --- | --- | --- | --- | --- | --- | --- | --- | --- | --- | --- | --- | --- | --- |
| Almeida et al. 2011 | Perth Metropolitan Area, Western Australia | Health in Men Study | Pseudo Population-based | Cross-Sectional | 3504 | 10^+^ | Subclinical | Male | 3473 | 31 | 0,0% | 75,3 | 4,1 | 69 - 87 | fT4, TSH | Diagnosis | No | 1,40 | 0,30 - 5,80 |  |
| Bensenor et al. 2016 | Brazil |  | Pseudo Population-based | Cross-Sectional | 12630 | 7 | Subclinical | Mixed | 12437 | 193 | 51,9% | 51,7 | 9,0 | 35 - 74 | fT4, TSH | Diagnosis | No | 1,60 | 0,88 - 2,88 |  |
| Chen et al. 2014 | Taiwan | NHIRD | Register | Cohort | 20975 | 9^+^ | Clinical | Mixed | 16780 | 4195 | 77,5% | 41,1 | 14,0 | ≥ 20 | Register | Register | Yes | 1,69 | 1,45 - 1,96 | * |
|  |  |  |  |  |  |  |  | Female | 13008 | 3252 | 100,0% |  |  |  |  |  |  | 1,66 | 1,41 - 1,97 | * |
|  |  |  |  |  |  |  |  | Male | 3772 | 943 | 0,0% |  |  |  |  |  |  | 1,81 | 1,24 - 2,67 | * |
| De Jongh et al. 2011 | Amsterdam | LASA | Pseudo Population-based | Cross-Sectional | 1120 | 7 | Subclinical | Mixed | 1089 | 31 | 48,2% | 75,5 | 6,6 | ≥ 65 | fT4, TSH | CES-D | No | 1,85 | 0,79 - 4,34 |  |
| Engum et al. 2002 | Nord-Trondelag County, Norway | HUNT-Study | Population-based | Cross-Sectional | 28830 | 9^+^ | Clinical | Mixed | 28303 | 181 | 66,7% |  |  | 40 - 89 | fT4, TSH | HADS-D | Yes | 1,09 | 0,72 - 1,66 |  |
|  |  |  |  |  |  |  | Subclinical | Mixed | 28303 | 346 |  |  |  |  |  |  |  | 1,07 | 0,79 - 1,46 |  |
| Hong et al. 2018 | Korea | KNHANES 2014 | Population-based | Cross-Sectional | 1704 | 8 | Subclinical | Mixed | 1658 | 46 | 51,5% | 44,3 | 0,6 | 19 - 76 | fT4, TSH | PHQ-9 | No | 7,05 | 1,67 - 29,67 |  |
| Ittermann et al. 2015 | West-Pomerania, Germany | SHIP, LEGEND | Population-based | Cohort | 1718 | 8^+^ | Clinical | Mixed | 1644 | 74 | n/A |  |  | 20 - 79 | TSH | Diagnosis | Yes | 4,09 | 1,18 - 14,15 | ° |
| Kim et al. 2010 | Gwangju, South Korea | 10/66 DIDCRP | Pseudo Population-based | Cross-Sectional | 458 | 8 | Subclinical | Mixed | 444 | 14 | 59,6% | 72,3 | 5,6 | ≥ 65 | TSH | GMS-B3 | Yes | 0,91 | 0,2 - 4,14 | # |
| Kvetny et al. 2015 | Naestved Municipality, Denmark | GESUS | Population-based | Cross-Sectional | 13521 | 8 | Subclinical | Mixed | 13236 | 285 | 53,0% | 53,5 | 13,3 | ≥ 20 | TSH | Diagnosis | Yes | 1,06 | 0,5 - 2,26 | # |
|  |  |  |  |  |  |  |  | Female | 6950 | 214 | 100,0% |  |  |  |  |  |  | 0,67 | 0,25 - 1,82 | # |
|  |  |  |  |  |  |  |  | Male | 6286 | 71 | 0,0% |  |  |  |  |  |  | 2,37 | 0,73 - 7,63 | # |

| Study | Location | Name | Population | Design | N | NOS | Sample | Gender | Euthyroid [n] | Thyroid Disorder [n] | Female [%] | Age mean [y] | Age SD [y] | Age range [y] | Assessment Thyroid Disorder | Assessment Depression | Thyroid Medication Intake | OR | 95% CI |  |
| --- | --- | --- | --- | --- | --- | --- | --- | --- | --- | --- | --- | --- | --- | --- | --- | --- | --- | --- | --- | --- |
| Manciet et al. 1995 | Gironde, France | PAQUID Survey | Pseudo Population-based | Cross-Sectional | 399 | 7 | Clinical | Mixed | 381 | 2 | 55,1% |  |  | ≥ 65 | fT4, TSH | CES-D | Yes | 1,48 | 0,07 - 31,30 | # |
|  |  |  |  |  |  |  | Subclinical | Mixed | 381 | 16 |  |  |  |  |  |  |  | 0,22 | 0,01 - 3,80 | # |
| Maugeri et al. 1998 | Catania, Eastern Sicily, Italy |  | Pseudo Population-based | Cross-Sectional | 60 | 6 | Clinical | Mixed | 50 | 10 | n/A |  |  | ≥ 70 | T3, T4, TSH | GDS-30 | Yes | 2,67 | 0,63 - 11,28 | # |
| Pop et al. 1998 | Eindhoven, Netherlands |  | Pseudo Population-based | Cross-Sectional | 558 | 8 | Clinical | Female | 540 | 3 | 100,0% | 49,9 | 2,2 | 47 - 54 | fT4, TSH | EDS | Yes | 3,45 | 0,60 - 19,80 |  |
|  |  |  |  |  |  |  | Subclinical | Female | 540 | 15 | 100,0% |  |  |  |  |  |  | 2,71 | 0,70 - 10,50 |  |
| Shinkov et al. 2014 | Bulgaria |  | Population-based | Cross-Sectional | 2287 | 7 | Subclinical | Mixed | 2198 | 89 | 55,1% | 47,7 | 14,5 | 20 - 84 | TSH | Zung SDS | Yes | 1,38 | 0,89 - 2,15 | # |
|  |  |  |  |  |  |  |  | Female | 1214 | 46 | 100,0% |  |  |  |  |  |  | 1,26 | 0,69 - 2,28 | # |
|  |  |  |  |  |  |  |  | Male | 984 | 43 | 0,0% |  |  |  |  |  |  | 1,79 | 0,9 - 3,55 | # |
| Thomsen et al. 2006 | Denmark | DNHR, DPCRR | Register | Cohort | 150960 | 8^+^ | Clinical | Mixed | 122770 | 28190 | 60,4% |  |  | ≥ 15 | Register | Diagnosis | Yes | 1,79 | 1,56 - 2,06 | # |
| Van de Ven et al. 2012 | Nijmegen, Netherlands | NBS-Study | Population-based | Cross-Sectional | 884 | 8 | Clinical | Mixed | 874 | 3 | n/A | 57 | 5,7 | 50 - 70 | fT4, TSH | BDI-Ia | No | 0,78 | 0,04 - 15,18 | # |
|  |  |  |  |  |  |  | Subclinical | Mixed | 874 | 7 | n/A |  |  |  |  |  |  | 2,19 | 0,42 - 11,40 | # |

Supplementary Table 1. Studies included in the present meta-analysis. Table legend:

* = converted from HR; ° = converted from RR; # = calculated from 2 x 2 table; + = included In RoB-Analysis

CES-D = Center for Epidemiologic Studies Depression Scale; HADS-D = Hospital Anxiety and Depression Scale; PHQ-9 = Patient Health Questionnaire 9; GMS-B3 = Geriatric Mental State Diagnostic Schedule; GDS-30 = Geriatric Depression Scale 30; EDS = Edinburgh Depression Scale; Zung SDS = Zung Self-Rating Depression Scale; BDI-Ia = Beck Depression Inventory Ia; Diagnosis = DSM- or ICD-conforming diagnosis of depression; NOS = Newcastle-Ottawa Scale

**Supplementary Table 2.1:** Additional Results of the Primary Analysis

| **Analysis** | **Hyperthyroidism** | |
| --- | --- | --- |
|  | crude | adjusted for possible reporting bias |
| Primary Outcome | 1.67 [1.49 - 1.87], p < 0.001  N = 15, I² = 6.4%, τ = 0.058 | N/A Egger's p = 0.964 |
|  | *Subgroup Analyses* | |
| Overt Hyperthyroidism (see Supplementary Table 2.2 for further analyses) | 1.70 [1.49 - 1.93], p < 0.001  N = 8, I² = 12.9%, τ = 0.069 | 1.67 [1.43 - 1.94], N = 1 Egger's p = 0.854 |
| Subclinical Hyperthyroidism (see Supplementary Table 2.2 for further analyses) | 1.36 [1.06 - 1.74], p = 0.015  N = 11, I² = 13.4%, τ = 0.151 | 1.28 [0.94 - 1.74], N = 2 Egger's p = 0.262 |
| Risk of Bias | 1.66 [1.40 - 1.97], p < 0.001 N = 5, I² = 41.6%, τ = 0.114 | N/A Egger's p = 0.853 |
| Female | 1.37 [0.91 - 2.05], p = 0.130 N = 3, I² = 46.7%, τ = 0.254 | 1.66 [1.16 - 2.39], N = 2  Egger's p = 0.154 |
| Male | 1.84 [1.34 - 2.54], p < 0.001 N = 3, I² = 0%, τ = 0.000 | N/A Egger's p = 0.416 |
| Population-based Studies | 1.53 [0.97 - 2.42], p = 0.066  N = 6, I² = 48.3%, τ = 0.362 | 1.36 [0.80 - 2.30], N = 1 Egger's p = 0.247 |
| "Pseudo" Population-based Studies | 1.74 [1.58 - 1.92], p < 0.001 N = 9, I² = 0%, τ = 0.000 | 1.75 [1.58 - 1.93], N = 1  Egger's p = 0.982 |
| Cohort Studies | 1.75 [1.58 - 1.95], p < 0.001  N = 3, I² = 3.6%, τ = 0.020 | 1.74 [1.52 - 2.00], N = 1 Egger's p = 0.280 |
| Cross-Sectional Studies | 1.39 [1.11 - 1.75], p = 0.004 N = 12, I² = 0%, τ = 0.000 | 1.34 [1.01 - 1.78], N = 2 Egger's p = 0.175 |
| Intake of Thyroid Medication | 1.63 [1.43 - 1.87], p < 0.001  N = 10, I² = 16.3%, τ = 0.082 | N/A Egger's p = 0.669 |
| No Intake of Thyroid Medication | 1.85 [1.20 - 2.87], p = 0.006  N = 5, I² = 0.3%, τ = 0.033 | N/A Egger's p = 0.760 |
| Clinical Diagnosis of Depression | 1.73 [1.57 - 1.91], p < 0.001 N = 6, I² = 0%, τ = 0.000 | 1.75 [1.58 - 1.93], N = 1 Egger's p = 0.855 |
| Score-based Diagnosis of Depression | 1.46 [1.07 - 1.99], p = 0.017 N = 9, I² = 10.1%, τ = 0.155 | 1.35 [0.89 - 2.04], N = 2 Egger's p = 0.185 |
|  | *Post-hoc Analyses* | |
| Older Population | 1.67 [0.93 - 3.03], p = 0.088 N = 5, I² = 0%, τ = 0.000 | 2.00 [1.19 - 3.35], N = 2 Egger's p = 0.637 |
| All Ages | 1.63 [1.38 - 1.92], p < 0.001 N =10, I² = 34.8%, τ = 0.133 | 1.58 [1.31 - 1.91], N = 1 Egger's p = 0.960 |
| Hypothyroidism | 1.36 [1.02 - 1.82], p = 0.037 N = 14, I² = 63.8%, τ = 0.373 | N/A Egger's p = 0.711 |
| Hyperthyroidism | 1.61 [1.34 - 1.93], p < 0.001 N =14, I² = 13.0%, τ = 0.124 | N/A Egger's p = 0.973 |

**Supplementary Table 2.2:** Additional Results regarding strictly overt and strictly subclinical Hyperthyroidism

Supplementary Table 2.1. Additional results of the primary analyses.

Effects are reported as OR and 95% confidence interval. N describes the number of studies included in the analysis. Egger's p-value is reported as two-sided, values <0.1 indicate potential publication bias.
Results reported as "crude" are not adjusted for potential publication biases.

| **Analysis** | **Overt Hyperthyroidism** | | **Subclinical Hyperthyroidism** | |
| --- | --- | --- | --- | --- |
|  | crude | assessed for bias | crude | assessed for bias |
| Primary Outcome | 1.70 [1.49 - 1.93], p < 0.001  N = 8, I² = 12.9%, τ = 0.069 | 1.67 [1.43 - 1.94], N = 1 Egger's p = 0.854 | 1.36 [1.06 - 1.74], p = 0.015  N = 11, I² = 13.4%, τ = 0.151 | 1.28 [0.94 - 1.74], N = 2 Egger's p = 0.262 |
| Low Risk of Bias | 1.65 [1.37 - 2.00], p < 0.001 N = 4, I² = 55.8%, τ = 0.132 | N/A Egger's p = 0.932 | 1.08 [0.8 - 1.46], p = 0.608 N = 2, I² = 0.00%, τ = 0.000 | N/A |
| Female | - | - | - | - |
| Male | - | - | - | - |
| Population-based Studies | 1.61 [0.60 - 4.30], p = 0.340  N = 3, I² = 50.1%, τ = 0.618 | N/A Egger's p = 0.683 | 1.36 [0.91 - 2.03], p = 0.130  N = 5, I² = 45.4%, τ = 0.286 | 1.16 [0.72 - 1.87], N = 2  Egger's p = 0.146 |
| "Pseudo" Population-based Studies | 1.75 [1.58 - 1.94], p < 0.001 N = 5, I² = 0%, τ = 0.000 | 1.75 [1.58 - 1.94], N = 1 Egger's p = 0.268 | 1.58 [1.04 - 2.40], p = 0.031 N = 6, I² = 0%, τ = 0.000 | 1.74 [1.17 - 2.58], N = 2 Egger's p = 0.253 |
| Cohort Studies | 1.75 [1.58 - 1.95], p < 0.001  N = 3, I² = 3.6%, τ = 0.020 | 1.74 [1.52 - 2.00], N = 1 Egger's p = 0.280 | N/A | N/A |
| Cross-Sectional Studies | 1.23 [0.84 - 1.80], p = 0.297 N = 5, I² = 0%, τ = 0.000 | 1.09 [0.70 - 1.69], N = 2 Egger's p = 0.302 | 1.36 [1.06 - 1.74], p = 0.015  N = 11, I² = 13.4%, τ = 0.151 | 1.28 [0.94 - 1.74], N = 2 Egger's p = 0.262 |
| Intake of Thyroid Medication | 1.69 [1.46 - 1.96], p < 0.001  N = 7, I² = 22.8%, τ = 0.089 | 1.66 [1.41 - 1.94], N = 2 Egger's p = 0.699 | 1.16 [0.92 - 1.46], p = 0.207 N = 6, I² = 0%, τ = 0.000 | 1.17 [0.93 - 1.48], N = 1 Egger's p = 0.877 |
| No Intake of Thyroid Medication | 0.78 [0.04 - 15.19], p = 0.870 N = 1, I² = 0%, τ = 0.000 | N/A | 1.91 [1.25 - 2.92], p = 0.003  N = 5, I² = 0%, τ = 0.000 | N/A  Egger's p = 0.364 |
| Clinical Diagnosis of Depression | 1.75 [1.58 - 1.95], p < 0.001  N = 3, I² = 3.6%, τ = 0.020 | 1.74 [1.52 - 2.00], N = 1 Egger's p = 0.280 | 1.37 [0.88 - 2.14], p = 0.164 N = 3, I² = 0%, τ = 0.000 | N/A Egger's p = 0.822 |
| Score-based Diagnosis of Depression | 1.23 [0.84 - 1.80], p = 0.297 N = 5, I² = 0%, τ = 0.000 | 1.09 [0.70 - 1.69], N = 2 Egger's p = 0.302 | 1.47 [1.01 - 2.15], p = 0.046 N = 8, I² = 35.1%, τ = 0.291 | 1.25 [0.81 - 1.93], N = 2 Egger's p = 0.304 |
|  | *Post-hoc Analyses* | | | |
| Older Population | 2.39 [0.65 - 8.82], p = 0.189 N = 2, I² = 0.00%, τ = 0.000 | N/A | 1.38 [0.72 - 2.64], p = 0.328 N = 4, I² = 0%, τ = 0.000 | 1.72 [0.91 - 3.23], N = 2 Egger's p = 0.044 |
| All Ages | 1.67 [1.41 - 1.98], p < 0.001 N = 6, I² = 34.8%, τ = 0.111 | 1.64 [1.36 - 1.96], N = 1 Egger's p = 0.990 | 1.42 [1.04 - 1.95], p = 0.029 N = 7, I² = 34.6%, τ = 0.237 | 1.24 [0.86 - 1.79], N = 3 Egger's p = 0.033 |
| Hypothyroidism | 1.69 [0.83 - 3.45], p = 0.152 N = 7, I² = 74.2%, τ = 0.709 | N/A Egger's p = 0.870 | 1.35 [1.05 - 1.73], p = 0.019 N = 11, I² = 58.6%, τ = 0.277 | 1.14 [0.88 - 1.47], N = 4 Egger's p = 0.003 |
| Hyperthyroidism | 1.66 [1.22 - 2.26], p = 0,001 N = 7, I² = 25%, τ = 0.198 | 1.59 [1.14 - 2.20], N = 1 Egger's p = 0.883 | 1.36 [1.06 - 1.74], p = 0.015 N = 11, I² = 13.4%, τ = 0.151 | 1.28 [0.94 - 1.74], N = 2 Egger's p = 0.262 |

Supplementary Table 2.2. Additional results of the subgroup analyses for clinical and subclinical hyperthyroidism.

Effects are reported as OR and 95% confidence interval. N describes the number of studies included in the analysis. Egger's p-value is reported as two-sided, values <0.1 indicate potential publication bias.
Results reported as "crude" are not adjusted for potential publication biases.

**References**

1. Zhang J, Yu KF. What's the relative risk? A method of correcting the odds ratio in cohort studies of common outcomes. *Jama* 1998; **280**(19)**:** 1690-1691.

2. Kessler RC, Chiu WT, Demler O, Merikangas KR, Walters EE. Prevalence, severity, and comorbidity of 12-month DSM-IV disorders in the National Comorbidity Survey Replication. *Arch Gen Psychiatry* 2005; **62**(6)**:** 617-627.

3. Shor E, Roelfs D, Vang ZM. The "Hispanic mortality paradox" revisited: Meta-analysis and meta-regression of life-course differentials in Latin American and Caribbean immigrants' mortality. *Soc Sci Med* 2017; **186:** 20-33.

4. Altman DG, Andersen PK. Calculating the number needed to treat for trials where the outcome is time to an event. *Bmj* 1999; **319**(7223)**:** 1492-1495.

5. Bland JM, Altman DG. The odds ratio. *BMJ* 2000; **320**(7247)**:** 1468.

6. Borenstein M, Hedges, L., Higgins, J., Rothstein, H. Comprehensive Meta-Analysis Version 3. Biostat: Englewood, NJ, 2013.
